# Consumer Perceptions of Precision Livestock Farming—A Qualitative Study in Three European Countries

**DOI:** 10.3390/ani11051221

**Published:** 2021-04-23

**Authors:** Caspar Krampe, Jordi Serratosa, Jarkko K. Niemi, Paul T. M. Ingenbleek

**Affiliations:** 1 Marketing and Consumer Behaviour Group, Department of Social Science, Wageningen University and Research, Hollandseweg 1, 6706 KN Wageningen, The Netherlands; paul.ingenbleek@wur.nl; 2 Research Park Universitat Autònoma de Barcelona, Universitat Autònoma de Barcelona, Av. De Can Domènech, 08193 Bellaterra, Spain; jordi.serratosa@uab.cat; 3 Bioeconomy and Environment, Natural Resource Institute Finland (Luke), Kampusranta 9, 60320 Seinäjoki, Finland; jarkko.niemi@luke.fi

**Keywords:** animal welfare, precision livestock farming, consumers, European cross-country comparison, focus group discussions, value chain, pigs, dairy cows

## Abstract

**Simple Summary:**

Precision livestock farming (PLF) will have a profound influence on animal husbandry, farm animal welfare and animal-based production chains. Even though consumers ultimately pay the costs of innovations, their perceptions of PLF technologies have been under-investigated. To explore consumer perceptions of PLF technologies within the pork and dairy value chains, altogether six focus group discussions were conducted in Finland, the Netherlands and Spain. The results indicate that consumers expect that PLF technologies will enhance the health and welfare of farm animals, while generating environmental improvements and increasing transparency in livestock farming. They, however, also expressed the fear that PLF technologies will lead to more industrialisation in livestock farming; that PLF technologies and data are vulnerable to misuse and cyber-crime; and that PLF information may be inadequately communicated to consumers. The findings are used to formulate directions for strengthening the acceptance of PLF technologies among European consumers.

**Abstract:**

Scholars in the fields of animal science and technology have investigated how precision livestock farming (PLF) can contribute to the quality and efficiency of animal husbandry and to the health and welfare of farm animals. Although the results of such studies provide promising avenues for the development of PLF technologies and their potential for the application in animal husbandry, the perspectives of consumers with regard to PLF technologies have yet to be the subject of much investigation. To address this research gap, the current study explores consumer perceptions of PLF technologies within the pork and dairy value chains. The investigation is based on results from six focus group discussions conducted in three European countries, each reflecting a different market environment: Finland, the Netherlands and Spain. The results indicate that consumers expect the implementation of different PLF technologies to enhance the health and welfare of farm animals, while generating environmental improvements and increasing the transparency of value-chain processes. The analysis further reveals three over-arching consumer concerns: (1) the fear that the integration of PLF technologies will introduce more industrialisation into livestock farming production; (2) the concern that PLF technologies and data are vulnerable to misuse and cyber-crime; and (3) the concern that PLF information is not communicated adequately to allow informed purchase decisions. The research findings provide directions for members of the animal-based food value chain to make informed decisions to improve their sustainability, social responsibility and credibility by endorsing the acceptance of PLF (technologies) amongst European consumers.

## 1. Introduction

The disruptive development of innovative precision livestock farming (PLF) technologies is expected to generate fundamental changes in animal husbandry. These technologies offer substantial opportunities for achieving a more holistic, evidence-based approach to monitoring and managing the health and welfare of farm animals and the productivity of livestock in real time [1,2]. The implementation of PLF technologies makes it possible to place cameras, microphones, temperature sensors, skin-conductivity sensors and/or augmented-reality tools sufficiently close to farm animals to enhance the monitoring and management of individual farm animals in real time [3,4,5]. Moreover, blockchain technologies make it possible to improve traceability and accountability across value chains, thereby allowing parties to trade in the absence of a mediator or trusting relationship [6,7]. At the same time, however, the development of PLF technologies raises new challenges with regard to the management, distribution and ownership of data [8,9]. These challenges could potentially have far-reaching effects on value chain processes and the stakeholders who are involved in them, including producers, intermediaries, retailers and consumers.

Previous studies have investigated how innovative PLF technologies can create benefits for and across farm animal species, production lines and countries [1,3,10,11]. Relatively little attention has been paid to the complex societal questions surrounding PLF technologies, including the perceptions of stakeholders with regard to the technologies applied and its associated data usage [12]. This research gap is particularly relevant to the large and heterogeneous group of consumers, which has often been neglected when investigating the adoption of innovative PLF technologies within farm animal value chains. The integration of consumer perceptions into research on PLF is important, as the success or failure of value chains that utilise PLF is ultimately determined by the purchasing decisions of consumers [13]. Moreover, the integration of consumer perceptions at an early stage of the innovation-development process has been shown to increase the societal acceptance of technological innovations [14]. Hence, a body of research exists that examines consumer perceptions of a wide variety of agricultural technologies [15,16,17,18], thus far not yet including PLF.

The objective of the current study is to investigate consumers’ perceptions of PLF technologies in pork and dairy value chains. Given that consumer research on PLF is still in the early stages, a qualitative research approach to gain in-depth understanding on consumer views towards PLF is appropriate. The investigation is therefore based on focus group discussions with consumers in three European countries: Finland, the Netherlands and Spain. This study contributes with important insights into the benefits and risks that consumers associate with the integration of PLF in the pork and dairy value chains, and reveal important factors influencing the further exploration and promotion of consumer acceptance of PLF technologies and products originating from value chains that utilise PLF technologies.

## 2. Materials and Methods

Focus group discussions, as a qualitative research method, have been used by researchers in a variety of disciplines (e.g., psychology, sociology, marketing and the social and behavioural sciences [19]), as they offer an opportunity to explore the perceptions that individuals have about specific topics or entities [20,21]. In a focus group discussion, a group of individuals of differing socio-economic and/or cultural backgrounds is assembled for the purpose of discussing a specific topic, with the goal of drawing insights from the complex personal experiences, beliefs, perceptions and attitudes of the participants through a moderated, interactive discourse [22,23,24,25,26,27]. To explore the research question, we selected heterogeneous samples of consumers in three countries (Finland, the Netherlands and Spain), thus accounting for variances between European consumers (from North to South) in terms of farm animal welfare [28] and the adoption and acceptance of innovative technologies [29,30,31]. Given that consumer perceptions in this regard are known to differ according to the species of farm animals [32], two focus group discussions were conducted in each country, one concentrating on the pork value chain and the other concentrating on the dairy value chain. To standardise the data collection procedure, a protocol to recruit participants and to carry out the focus group discussions was prepared.

### 2.1. Participants

Given that the use of PLF technology is still in the emerging phases and the products are expected to reach the food market more widely in the near future, we invited participants younger than the mean age of the principal shoppers in the country, as well as innovative early adapters of technological developments (participants were screened according to the technology adoption scale, selecting participants with scores of at least 15 up to a maximum of 25 points [33]). Participants who indicated that they did not consume animal-based products or that they were employed in the areas of marketing, research, media, journalism or the food industry were excluded from participation, as their expert opinions were likely to influence the contributions of other participants.

Following the selection criteria, in Finland and the Netherlands participants were recruited via market research companies specialised in qualitative research (www.makery.fi in Finland and www.bureaufris.nl in The Netherlands). In both countries, participants received a monetary incentive of EUR 60 and 75, respectively, for their participation. In Spain, participants were recruited via a university intern consumer panel, offering participants personal developmental programs as an incentive for their participation. The recruitment procedure resulted in a total of 56 participants (30 female, 26 male) for the focus group discussions in the three European countries. In the Netherlands, the groups consisted of 16 participants (8 for the pork group and 8 for the dairy group; 3 male and 5 female). Their average age was 31.16 years (*SD* = 7.29), and the average innovativeness score of both groups was 21.88 (*SD* = 2.58). In Spain, there were 20 participants (10 for the pork group and 10 for the dairy group; 4 male and 6 female), with an average age of 31.55 years (*SD* = 8.33) and an average innovativeness score of 18.15 (*SD* = 2.78). The discussions in Finland included a total of 20 participants (10 for the pork group and 10 for the dairy group; 5 male and 5 female), with an average age of 39.60 years (*SD* = 8.33) and an average innovativeness score of 17.3 (*SD* = 2.94) (see Table 1). The participants in the three countries had comparable profiles of profession.

To ensure that the consumers participating in the pork or dairy focus groups did not differ significantly from one another in terms of their attitudes towards animals (as measured according to the Animal Attitude Scale [34]), the tendency to search for innovative products (according to the novelty-seeking scale [35]) and the need for cognitive stimulation (according to the need for cognition scale [36]), each participant was asked to complete a post-discussion questionnaire. The results of this questionnaire did not reveal any significant differences between the participants of either the pork or dairy focus group discussions. This suggests that the prevailing attitudes of the participants towards animals, their tendency to seek innovation and their need for cognitive stimulation can be neglected when interpreting the qualitative data generated by the discussions.

We expected differences between the participants from different countries. To demonstrate these differences, the same validated scales used in the post-questionnaire were analysed. As expected, the results reveal that participants from the three European countries differ with regard to their attitudes towards animals and their need for cognition. The animal-attitude scores of Finnish participants (*M* = 17.89, *SD* = 2.33) were significantly lower than those of the Dutch (*M* = 19.50, *SD* = 2.50) and Spanish (*M* = 19.95, *SD* = 1.55) participants. In contrast, Spanish participants (*M* = 11.84, *SD* = 2.69) tended to require significantly less information than did either Dutch (*M* = 13.75, *SD* = 1.69) or Finnish participants (*M* = 15.16, *SD* = 2.27). These differences accounted for the expected variance between the three European countries (from North to South) [28,29,30,31].

### 2.2. Focus Group Procedure

To harmonise the data collection across the three countries, we used a pre-defined protocol to conduct the focus group discussions. Before each discussion started, an experienced moderator explained the general objective and methodological approach of the study and asked consumers to sign an informed consent form, thereby confirming their voluntary participation in the focus group discussions, as well as their permission for the discussion to be recorded, in accordance with the Declaration of Helsinki and the General Data Protection Regulation of the European Union. After all participants had provided their consent, the discussions began. After stating their initial impressions with regard to PLF, the participants received a brief explanation of PLF, based on a pictorial graph and technological examples (see Supplementary Material 1). In addition, the lifecycles of pigs and dairy cows were displayed (see Supplementary Materials 2 and 3), in order to provide a general understanding of the specific steps in the pork or dairy value chain, thereby allowing participants to transfer their knowledge, ideas and concerns to the specific research questions. The specific research questions were subsequently asked, according to the pre-defined protocol [37]. Finally, the participants were requested to complete a short post-discussion questionnaire.

Each focus group discussion lasted for 150–180 min, depending on the dynamics, the number of participants and the country. To control for time-dependent events (e.g., a media report on the possible mis-management of farm animals), all six of the discussions were conducted during the same week in January 2020.

### 2.3. Topics and Questions Addressed

Based on a literature review, three topics that could be expected to be of relevance with regard to shaping consumer perceptions of PLF technologies were identified. These topics represent the core issues of the focus group discussions.

First, previous research identified that the consumers’ knowledge, pre-existing attitudes, the risk and benefit appraisal and trust in the information source fundamentally influence consumers’ perceptions of (technological) agricultural innovations [15,16,17,18]. In the case of disruptive technological developments, such as PLF, the identified influencing factors are usually impossible for consumers to assess, which in the past has often led to strong resistance from a significant number of consumers [14,38,39]. It is, hence, of crucial importance to identify the benefits and fears that consumers perceive in relation to PLF technologies. Only when stakeholders within value chains have insight into the concerns and demands of consumers can they actively target efforts in order to foster the acceptance of PLF technologies. For this reason, the first phase of the focus group discussion concentrated on the technological, institutional and environmental opportunities and constraints that consumers identified in relation to PLF (and associated technologies). This was done in order to explore factors that influence the likelihood that consumers will either accept or reject PLF technologies. For this reason, participants in all three countries and in both farming sectors were asked to answer the following question: *In your opinion, what are the technological, institutional and environmental opportunities and constraints of PLF technologies?*

Second, as indicated before, the implementation of PLF technologies is expected to have a fundamental impact on animal husbandry, farm animals and various processes and stakeholders within the value chain [1,2,8,9]. It is, therefore, vital to investigate what consumers believe with regard to how PLF (and the associated technologies) will transform animal and livestock farming in the future. The second topic of the focus group discussion examined the expected and future benefits and drawbacks associated with the implementation of PLF technologies, along with the repercussions for production processes in the dairy and pork value chains, based on the following questions: *To what extent do you think PLF will transform animal husbandry? How could the utilisation of PLF (and the associated technologies) lead to improvements for society/farm animals/economic situations?*

Third, product communication is a crucial aspect for the sale of products produced using PLF [40]. To minimise information asymmetries between consumers and other stakeholders within the value chains, various information approaches to inform consumers about the credibility of product (credence) attributes have been used in the past. The classic approach to informing consumers involves ‘signalling’, with the help of labels. As indicated by the gap between attention and behaviour [41], however, these label approaches have not been very successful in responding to the dynamic needs of consumers for information about specific product attributes (e.g., green consumption or animal welfare) [42]. It is therefore important to understand how consumers obtain information when making purchase decisions, as well as to explore the possible role played by innovative PLF technologies in addressing information asymmetries within value chains and their stakeholders [43]. Accordingly, the third topic of the focus group discussions addressed the consumers’ demand for and ideas about communication approaches relating to PLF (associated technologies), based on the following questions: *How do you inform yourself about livestock products at the point of sale? To what extent do labels influence your purchase decisions? In your opinion, how should products produced using PLF look like? What would be the ideal way to present information to you about products produced using PLF?*

### 2.4. Data Analysis

The content of the recorded, qualitative data from all three European countries was thematically transcribed, coded and analysed by experienced researchers in each country. Summary notes of the observations and impressions were also integrated into the data analysis. In order to guarantee an objective treatment of the collected data, the data were reviewed and compared by at least two researchers in each country (who all had experience with qualitative data analysis). More specifically, a content-analytic technique was applied in order to extract information about the identified topics [37]. After the individual country-level analysis, the results and conclusions drawn from the data analysis were shared with the team of researchers from all three countries. In a joint workshop, the results and conclusions of the focus group discussions were examined, discussed and compared by topic, and similarities between the research findings from the three countries were identified. Finally, conclusions were drawn.

## 3. Results

When confronted with the terminology of PLF, participants in the focus groups for both farm animal species in all three countries indicated that they had ‘*never heard*’ about PLF and its associated technologies. After introducing the concept of PLF to the participants, they understood its repercussions, such that they were able to think deeply about the research questions, transferring their knowledge to the identified topics. Interestingly, only minor differences could be identified between the pork and dairy groups with regard to the participants’ perceptions of PLF, and several marginal differences were identified between participants in the three countries. The specific research findings associated with the identified topics are depicted below, following the same topic-wise structure discussed in Section 2.3, indicating the benefits and fears of the participants, along with the transformational impact and the communication practices associated with the implementation of PLF technologies.

### 3.1. Consumer Perceptions of PLF: Benefits and Fears

Participants in each of the three countries identified several benefits and fears associated with the integration of PLF technologies within the respective value chains. In particular, they reported expecting that PLF technologies would have the ability to improve the transparency of livestock-farming practices within the value chains, as illustrated by the following quotations:

- ‘*[PLF data makes the] lives of pigs transparent*’.
- ‘*[PLF technologies provide] faster and detailed information about total effects in the big picture* [*of livestock farming*]’.

As a result of the improved transparency, PLF technologies were expected to ‘*increase the [consumers’] control of the whole production*’, given that PLF data can be provided to ‘*everyone*’, and ‘*if you [as a producer] have nothing to hide, why not provide all the relevant information [concerning farm animals?]*’. The participants further predicted that PLF technologies would enhance food safety within the value chains, and that it would ‘*increase [farm] animal welfare*’ and health and save the environment (e.g., by producing less CO_2_ emissions). The participants’ belief that PLF would reduce CO_2_ emissions is connected to the observation that PLF technologies would reduce ‘*odour and noises*’ around the farms. Participants in all three countries expected that advancements relating to PLF and process optimisation would enhance product quality and improve the health and welfare of farm animals. Given the impact that these outcomes are assumed to have on the pricing of PLF products, the participants reported expecting that products produced using PLF would be more expensive in the short term (due to high investment costs). In the longer term, however, they predicted that prices would decline once PLF technologies become integrated within the value chains (thereby becoming the new normal), thus allowing consumers from all socio-economic backgrounds to purchase PLF products.

Whilst the participants expected the integration of PLF (and the associated technologies) to bring a variety of benefits, they also expressed fears with regard to the implementation of PLF (including both technologies and data). Directly related to the notion that PLF (and the associated technologies) can optimise livestock farming, participants from all three countries noted that the implementation of PLF would carry the risk that livestock farming would become more ‘*robotised and digitalised*’. The participants expressed particular concerns that PLF (and the associated technologies) would ‘*replace farmers’ workplaces with machines*’, thus shifting the ‘*power*’ away from farmers towards a more ‘*industrialised [technology-based] production]*’, with less ‘*human attention to farm animals*’. The concerns about the negative consequences of PLF for farm animals and the stakeholders working with them can be illustrated by the following quotation: ‘*Farmers became farmers for a reason; otherwise they [would] have become computer engineers*’. Following up on this thought, participants also expressed the concern that PLF technologies are vulnerable to cyber-attacks, data leaks and/or misuse. These findings suggest that the integration of such technologies is highly dependent upon the digital and energy infrastructure needed to implement and apply them. With regard to the data generated by PLF, the participants expressed concerns with regard to how trustworthy such data would be, asking ‘*who is in charge of the data?*’, given that ‘*data is knowledge’* and ‘*knowledge is power*’ (Table 2).

Although participants from all three countries shared the aforementioned benefits and fears associated with the integration of PLF technologies, several differences in opinions were identified between the participants from the various countries. For example, Finnish participants expected that PLF (and the associated technologies) would reduce the workload of farmers and other stakeholders within the value chain, and that this would ultimately translate into positive outcomes for consumers, as reflected in reduced prices for PLF products. Dutch and Spanish participants were convinced that the integration of PLF technologies would be expensive and that it would generate more administrative work for farmers, with regard to analysing the data collected through PLF (this view ignores the possibility of automatic data analysis), thus ultimately increasing product prices. Although participants from all three countries expressed privacy concerns with regard to PLF technologies and data, those from the Netherlands were particularly concerned about privacy issues in relation to the disclosure of PLF data.

### 3.2. Consumer Perceptions of PLF: Transformational Impact on Livestock Farming

Participants in all three countries agreed that the integration of PLF would generate ‘*big chances*’ in livestock farming. With respect to animal welfare, participants expected that PLF technologies would improve farm animal health, detecting diseases earlier than conventional methods. These technologies were thus expected to serve as a tool for forecasting and ‘*control*’, thereby preventing situations that could endanger farm animals, whilst also controlling and monitoring the health and welfare status of individual farm animals at any given time. The participants further referred to the notion that data generated through PLF could be used to inform consumers about ‘*the [good] life of the animals*’ and to create a ‘*guide of the best [practices for] pig producers*’ in order to enhance ‘*excellence*’ in practice, improve animal welfare and enhance the quality of products produced through PLF (similar to the ‘Michelin’ guide, which awards up to three Michelin stars to hotels and restaurants for excellence in cuisine (https://guide.michelin.com)).

With regard to the actual production processes and stages of farm animals, the participants expected that PLF would affect current practices once the technologies have been integrated in the value chains. In the reproduction stage of pigs and heifers, the participants expressed the expectation that PLF and the associated technologies would foster genetic control in order to improve breeding practices, thereby resulting in healthier and better performing farm animals. Interestingly, although they expected that the integration of PLF technologies would reduce the ‘*stress*’ of animals in the process of rearing of pigs, they expected that it would increase ‘*stress*’ in dairy cows, as ‘*the cow’s life would be data-driven*’, thereby ‘*reducing interactions with farmers*’.

With regard to *transportation and slaughtering procedures*, the participants expressed the expectation that PLF technologies would help to ensure that farm animals would not suffer during transportation (e.g., by regulating the feed and water supply), thus allowing for the ‘*ethical transportation treatment*’ of farm animals. Moreover, they expected that PLF technologies could ensure that farm animals would ‘*not suffer*’ during the slaughtering process, for example by measuring whether the animals are properly anaesthetised before slaughter.

The participants expressed the expectation that the distribution and marketing of PLF products would benefit from the integration of related technologies within both farming sectors. With regard to pork production, participants expected PLF and the data that it would generate to provide evidence of particular product attributes (e.g., sanitary control, quality aspects and/or food safety), which could subsequently be communicated to consumers. In the dairy value chain, participants expected that PLF would make it possible to predict the exact amount of milk needed by the market. This was further expected to have direct consequences for the value chain (e.g., in terms of logistics/transportation costs).

### 3.3. Consumer Perceptions of PLF: Is It Purely a Matter of Communication?

Participants in all three countries indicated that labels continue to be the primary source of information about animal-based products at the point of sale. At the same time, however, they indicated that the information displayed through labelling approaches is often perceived as ‘*confusing*’ and/or untrustworthy, given that it is ‘*provided by big companies*’, which tend to be perceived as less trustworthy. Participants indicated that product information should be displayed in an ‘*easily accessible, recognisable, understandable and attractive manner*’. Several product attributes were identified as being of crucial importance to the evaluation of innovative pork or dairy products: (i) health and food-safety monitoring, (ii) production system, (iii) origin of farm animals and (iv) possibility of visiting the farm. Interestingly, participants expressed reluctance to buy meat products online, as they preferred to ‘*see*’ and ‘*feel*’ them with their ‘*own eyes and hands*’, such that they could evaluate these product attributes themselves.

As indicated above, participants identified trust in the information provided (and its source) as an important aspect for consumers. Participants indicated that they would trust PLF and the associated technologies if the data were to be validated by a trustworthy organisation. Conversely, they would not trust PLF data that were managed by companies that could be expected to care solely about their own economic benefits. The participants in the three countries differed with regard to their reasons for trusting or not trusting animal-based products. For example, Finnish participants identified the origin of the product as an important product attribute, which should be indicated on or with a product. Spanish participants reported that they do not evaluate all of the details provided on labels, instead using only a few (label-based) information cues. Dutch participants interpreted new labels in relation to existing labelling approaches. Although participants from all three countries identified labels as an important source of information, they also noted that the information provided by static label approaches is not sufficient, and that future labelling approaches should go beyond the informational level of existing, ‘*traditional*’ animal-based labels, including the possibility to obtain information through innovative, dynamic communication technologies.

## 4. Discussion and Implications

As indicated by the findings of this qualitative study, it is of crucial importance for researchers and practitioners to consider the opportunities and concerns identified by consumers as early as possible in the development and implementation of innovative PLF technologies [14]. To date, this perspective has been largely ignored, even though consumers are unlikely to accept the utilisation of products produced through PLF until they perceive such technologies as legitimate [44]. In this light, the focus group discussions conducted in this study identified a number of opportunities presented by PLF technologies, as well as three over-arching concerns that should be considered by both researchers and practitioners when implementing PLF technologies: (1) the fear that the integration of PLF technologies will introduce more industrialisation, digitalisation and robotisation into livestock farming production; (2) the concern that PLF data can be manipulated and/or that cyber-criminals can gain unauthorised access to PLF systems; and (3) the concern that PLF information will not be communicated adequately to consumers, thereby ignoring their need for personalised information on which to base their purchase decisions.

### 4.1. The Fear of Industrialisation, Robotisation and Digitalisation

Participants in the focus group discussions expressed the fear that the integration of PLF technologies will introduce more industrialisation, digitalisation and robotisation to the livestock farming production. Such beliefs fuel the fear that robots, machines and/or algorithms will ultimately be capable of replacing humans in their daily working routines once they reach a certain level of effectiveness. This would have several negative consequences, as it could reduce ‘*human attention*’ to farm animals, thereby reducing the level of emotional attachment to farm animals and decreasing the welfare of both humans and animals. This finding is also consistent with the OneWelfare approach, e.g., [45], which emphasises the interconnectedness of animal welfare, human wellbeing and the conservation of the environment as a whole. Moreover, the consumer concerns identified in this study are consistent with the results of studies on the practices used to manage the health and welfare of farm animals, which emphasise consumer preferences for natural, non-invasive approaches to manage farm animals, e.g., [46].

The fluctuating risk–benefit analyses of consumers with regard to the ways in which PLF is likely to influence animal-based production chains, farm animals and relevant stakeholders thus reflect a dilemma. This is largely because although consumers are willing to accept innovative PLF technologies (when the perceived personal benefits outweigh the perceived risks [47]), they tend to be critical, or even afraid, of PLF technologies, as they are perceived to place humans in competition with robots and computers for employment and other fundamental resources [48,49]. This finding has implications for consumer acceptance of PLF technologies, because it offers an addition to the frequently mentioned influencing factors, such as consumers’ prior knowledge [15], their attitudes towards the technology [16], the transparency of a technology [50], the behavioural control [51] and the (institutional) trust associated with a technology [18]. Accordingly, when integrating PLF technologies, it would be advisable to educate consumers about the personal benefits associated with PLF technologies, in order to allay their possible fears [52]. Stakeholders should also present evidence of these benefits, focusing on existing achievements that may increase consumer trust in PLF innovations (e.g., by highlighting the advantages of PLF technologies for the health and welfare of animals or the increased transparency of processes within the value chain). In this regard, however, it is important to consider country-specific differences. For example, stakeholders could highlight country-specific data-protection strategies when using PLF technologies, thereby addressing the needs of consumers in specific countries.

### 4.2. The Concerns Related to Cyber-Crime and Data Misuse

Participants in the focus groups expressed the concern that PLF technologies and data could be manipulated by stakeholders within the value chain or by external parties seeking economic benefits. They also referred to the risk that cyber-criminals could gain unauthorised access to PLF systems. These concerns are also expressed by other value chain stakeholders [53,54]. The consumers’ concerns may be a result of the complexity of PLF technologies and data, which makes it particularly challenging for consumers to understand how these innovative technologies and the data that they collect can assist animal-based value chains and their stakeholders. In light of this complexity, consumers form their opinions about PLF technologies based on their “gut feelings”, which results in so-called heuristic-based information processing and judgments [14]. These aspects seem to be greatly influenced by the consumers’ individual characteristics [14]. The perceived vulnerability of PLF technologies and the hypothetical misuse of PLF data were therefore identified as additional fundamental challenges in the implementation of PLF technologies. To address these consumer concerns and to guarantee the validity, reliability and trustworthiness of PLF technologies (and particularly the data generated by these technologies), consumers should be informed about the security measures that have been taken to guarantee the security and validity of PLF data. Unless consumers understand and perceive such measures as trustworthy, they will not have confidence in the information generated by PLF technologies [14,55]. Consequently, the end-users of the data (e.g., retailers, intermediaries and producers) may consider cooperating with trustworthy, independent institutions, which could help to secure and validate the data obtained through the use of innovative PLF technologies, as perceived by consumers. These partners could be independent institutional bodies (e.g., governmental agencies or non-profit organisations), or they could be developed through innovative technological solutions (e.g., blockchain technology). Regardless of the measures implemented to validate PLF technologies and data, consumers need to perceive them as legitimate [44,56]. The European Code of Conduct for agricultural data sharing by contractual agreement may be a first step in the right direction, as it strengthens trust between value chain stakeholders and consumers [57]. In a similar manner, secured and validated PLF data could be used to provide information about ‘excellence’ in production practices, thereby generating opportunities for business innovations, innovative products, brands or even production systems.

### 4.3. The Concerns Relating to Inadequate Communication Systems

The participants understood that the implementation of PLF practices and the associated potential for collecting and exploiting innovative data could offer a solution to their fundamental need for specific, personalised information about farm animals. In the absence of alternatives, however, most of the participants noted that they currently retrieve product information from product labels or by asking sales representatives for additional information. The participants perceived the advancing technological development within the field of information system and communication (e.g., social media, online shopping, retail innovation) and the utilisation of existing innovative technological solutions to provide validated, real-time PLF data as beneficial, as these developments addressed their dynamic need for information. These results support current voices that consider innovative (communication) technology to be at the core of modern retailing [58]. Participants therefore called for more holistic and dynamic approaches to communication, whilst noting that the utilisation of the most commonly applied static label approaches (the informational ‘push principle’) is insufficient. Accordingly, the participants also called for the organisation (or re-organisation) of existing communication approaches according to an informational ‘pull principle’, which would allow consumers to request and receive trustworthy information about specific product attributes, PLF production systems or PLF technologies, depending on their personal situations, the environmental context or their product-related involvement [59]. Such an application would allow stakeholders within the value chain (e.g., retailers) to cope with the heterogenous demands for more and detailed information about animal-based products (as expressed by the European consumers participating in this study), thereby helping consumers to make well-considered purchase decisions based on considerations of animal welfare [60]. The development of dynamic and personalised consumer applications based on PLF technologies and data could therefore allow consumers to specify a priori informational demands for animal-based products. Real-time PLF data could then be aligned to the indicated specifications, thus displaying/suggesting only products that would meet the indicated consumer preferences, thus reducing the complexity faced by consumers when taking decisions on animal-based products at the point of sale, regardless of the shopping environment (online or offline).

## 5. Conclusions

Focus group participants from three European countries expressed the expectation that the integration of PLF technologies within the system of farm animal production would lead to improvements in the health and welfare of farm animals and the environment, in addition to enhancing the transparency and control of processes and stakeholders within the value chain. The participants also identified three over-arching consumer concerns: the fear that the integration of PLF (and the associated technologies) will lead to more industrialisation, digitalisation and robotisation in livestock-farming production systems; the vulnerability of innovative PLF technologies and the risk that PLF data will be misused, and the concern that PLF information is not communicated adequately. Researchers and practitioners should take the identified concerns and the cross-cultural differences into account when implementing PLF technologies, in order to explore and foster consumer acceptance of PLF technologies across Europe. Meanwhile, differences in consumer perceptions of PLF between the pork and dairy value chains seem to play a subordinate role.

The present qualitative research findings help to fill the research gap on consumer perceptions of PLF technologies and provide guidance on how to address the identified consumer concerns about PLF technologies. Future studies should elaborate on the consumer perspectives in greater detail. In order to cover the vast and heterogenous group of consumers and farm products, a quantitative research approach might be the logical next step in this research process. Such an approach might also incorporate countries from the ‘West to East’ of the European Union.

## Figures and Tables

**Table 1:** Demographic and psycho-social characteristics of the consumers participating in the focus groups conducted in Spain, The Netherlands and Finland.

Country/Criteria	Spain	Netherlands	Finland	Total
**Number participants**	*N =* 20 (Dairy *n =* 10; pork *n =* 10)	*N* = 16 (Dairy *n =* 8; pork *n =* 8)	*N =* 20 (Dairy *n =* 10; pork *n =* 10)	*N =* 56 (Dairy *n =* 28; pork *n =* 28)
**Mean Age of Participants**	Dairy focus group *M*_age_ = 30.40; *SD* = 7.93 Pork focus group *M*_age_ = 32.70; *SD* = 8.73	Dairy focus group *M*_age_ = 28.25; *SD* = 7.94 Pork focus group *M*_age_ = 34.13; *SD* = 6.64	Dairy focus group *M*_age_ = 39.60; *SD* = 9.35 Pork focus group *M*_age_ = 39.60; *SD* = 7.31	Dairy focus group *M*_age_ = 33.07, *SD* = 8.41 Pork focus group *M*_age_ = 35.57, *SD* = 7.56
**Innovative Attitude Scale**	Dairy focus group *M_i_*_nnovativeness_ = 17.90; *SD* = 2.28 Pork focus group *M*_innovativeness_ = 18.40; *SD* = 3.27	Dairy focus group *M*_innovativeness_ = 21.88; *SD* = 2.58 Pork focus group *M*_innovativeness_ = 21.88; *SD* = 2.58	Dairy focus group *M*_innovativeness_ = 17.1; *SD* = 2.85 Pork group M_innovativeness_ = 17.5; *SD* = 3.03	Dairy focus group *M*_innovativeness_ = 18.7; *SD*= 2.57 Pork focus group *M*_innovativeness_ = 19.07; *SD* = 2.96

**Table 2:** Benefits and risks associated with the integration of PLF and the associated technologies, as identified by focus group participants.

Benefits	Risks
Increased transparency in the value chain and its processes	Increased digitalisation and robotisation in animal farming
Improved health and welfare of farm animals	Decreased ‘human attention’ to farm animals→decreased animal welfare
Environmental improvements: less emissions	Vulnerability of PLF technologies and data leaks
Improved productivity and control of production processes	Highly dependent on the digital and energy infrastructure and supply
Improved food safety	Lack of trust in the management of PLF data
Pain-free slaughtering	More administrative work for stakeholders in the short term
More freedom for farmers	More technological waste

## Supplementary Materials

The following are available online at https://www.mdpi.com/article/10.3390/ani11051221/s1: Supplementary Material 1. Precision livestock farming; Supplementary Material 2. Lifetime cycle of pigs; Supplementary Material 3. Lifetime cycle of dairy cows.
