# Supplementary material for: Consumer Perceptions of Precision Livestock Farming—A Qualitative Study in Three European Countries
Source: Animals (Basel). 2021 Apr 23;11(5):1221. doi: 10.3390/ani11051221 (PMC8146409; doi:10.3390/ani11051221)
Supplement: Supplementary file 1 [file animals-11-01221-s001.zip › Supplementary Material/Supplementary material 1.pdf]

Precision livestock farming

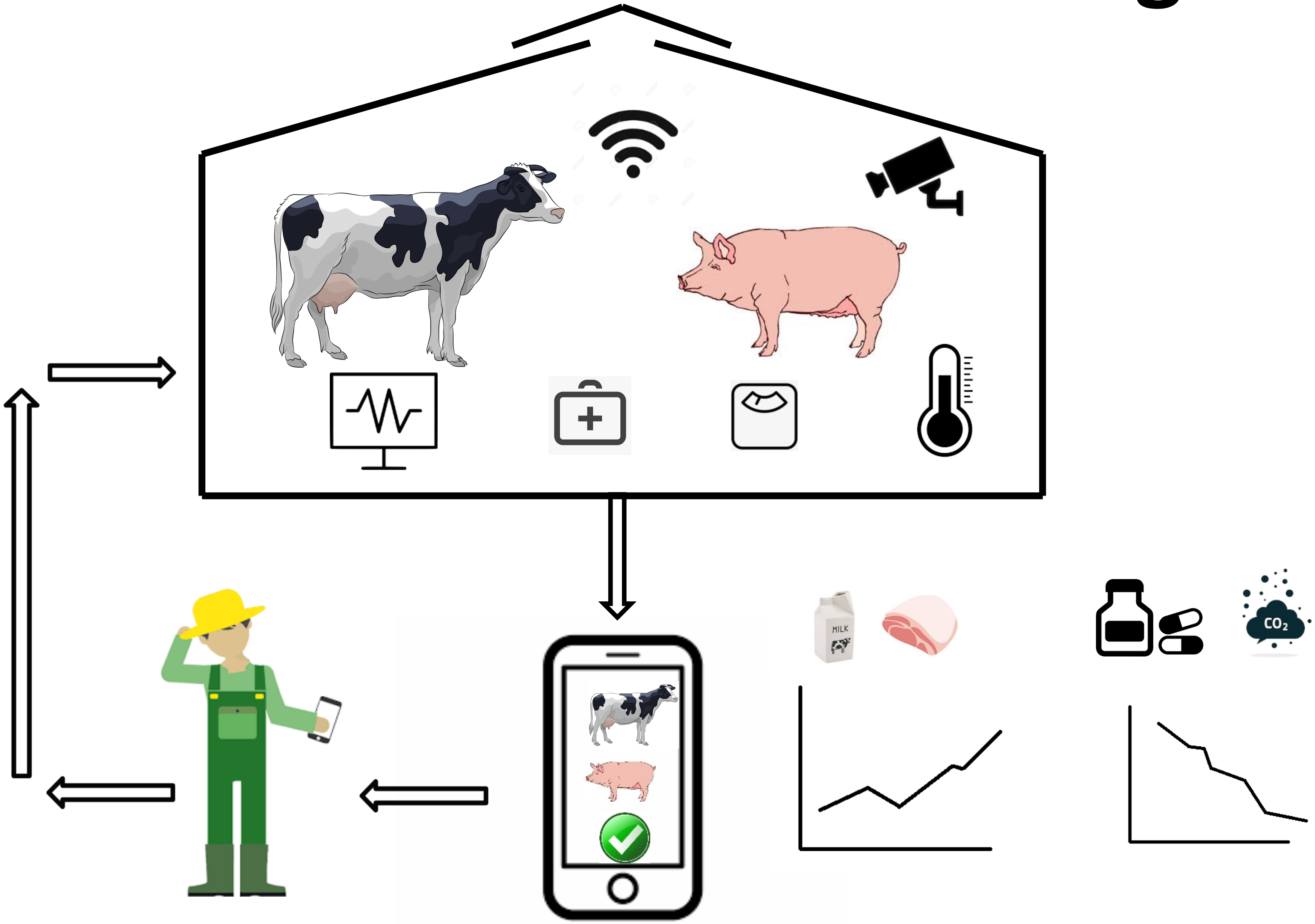

Precision livestock farming aims to 1) collect data on, for example, animal activity, health, body weight, amount of produced milk, conditions in a barn (on the graph we refer to animals in a barn), 2) process this data into USEFUL INFORMATION (on the graph we refer to mobile phone and a farmer), in order to 3) IMPROVE HERD MANAGEMENT (on the graph we refer to plots). USEFUL INFORMATION is for example the ID of a cow, which is moving less than expected or location of a pig, which is growing slower than expected. IMPROVED HERD MANAGEMENT means that a farmer is able to obtain more milk or meat produced but also decrease antibiotic consumption and greenhouse gas emission or improve animal welfare.
