# Supplementary figures and images for: Consumer Perceptions of Precision Livestock Farming—A Qualitative Study in Three European Countries
Source: Animals (Basel). 2021 Apr 23;11(5):1221. doi: 10.3390/ani11051221 (PMC8146409; doi:10.3390/ani11051221)

# Lifetime cycle of a pig

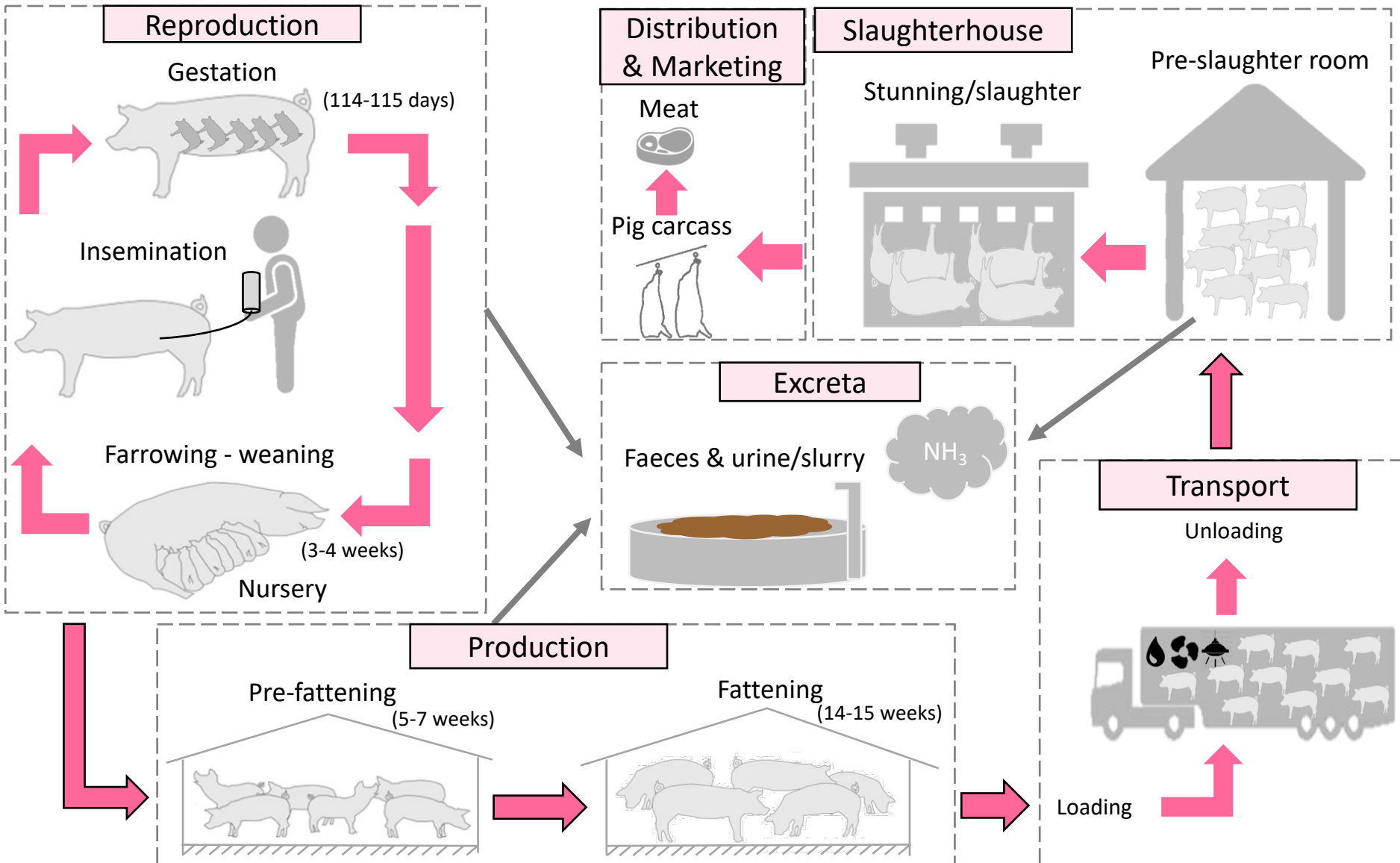

Supplement: Supplementary file 1 [file animals-11-01221-s001.zip › Supplementary Material/Supplementary material 2.pdf]

# Lifetime cycle of dairy cow

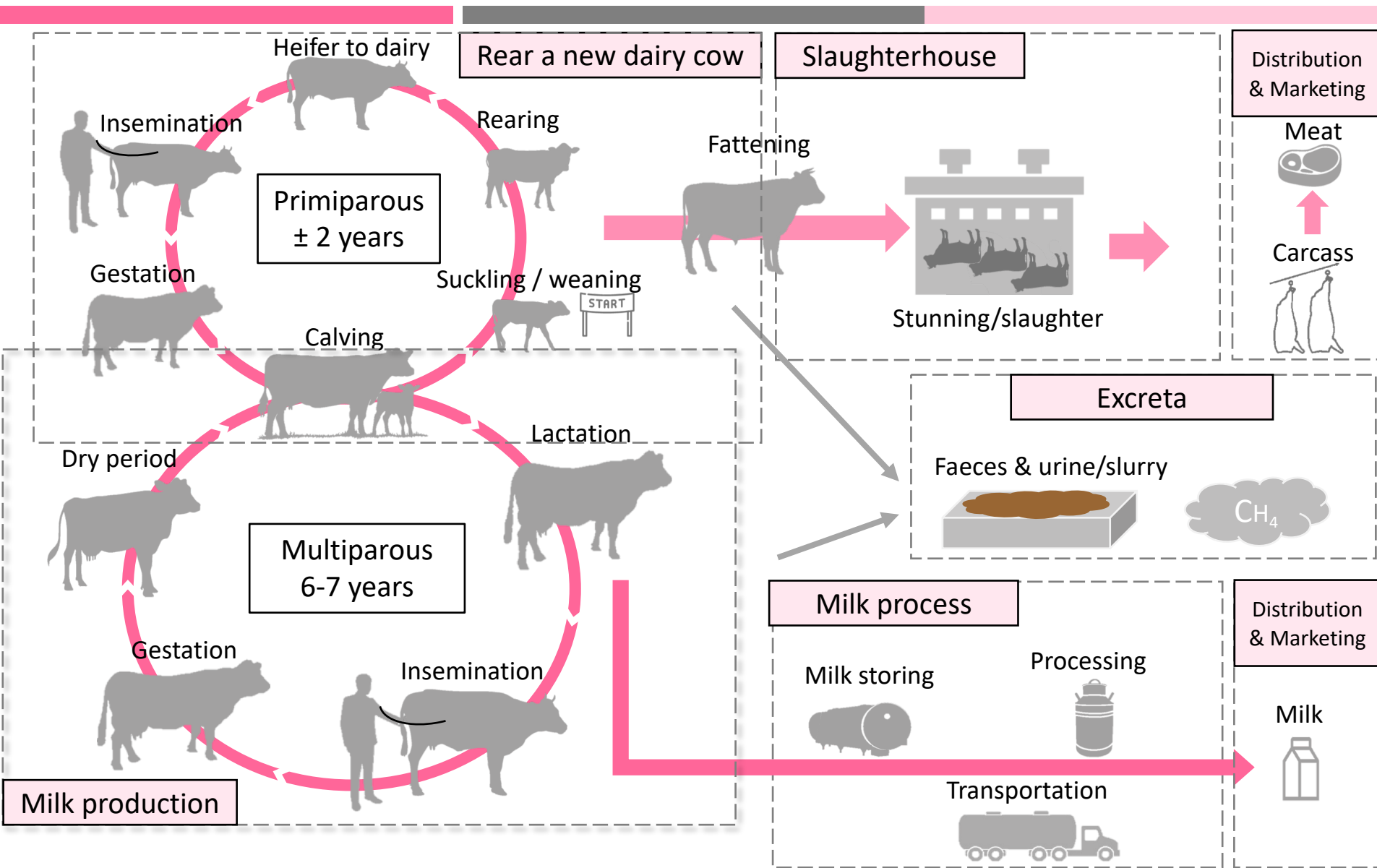

Supplement: Supplementary file 1 [file animals-11-01221-s001.zip › Supplementary Material/Supplementary material 3.pdf]
